# Similarities and differences in people accessing prevention and recovery care services and inpatient units in Victoria, Australia

**DOI:** 10.1186/s12913-020-05402-3

**Published:** 2020-06-16

**Authors:** Georgina Sutherland, Carol Harvey, Holly Tibble, Matthew J. Spittal, John Farhall, Justine Fletcher, Graham Meadows, J. Richard Newton, Ruth Vine, Lisa Brophy

**Affiliations:** 1 Melbourne School of Population and Global Health, University of Melbourne, Parkville, VIC Australia; 2 Psychosocial Research Centre, Department of Psychiatry, University of Melbourne, Parkville, VIC Australia; 3 Usher Institute, University of Edinburgh, Edinburgh, Scotland; 4 Department of Psychology and Counselling, La Trobe University, Bundoora, VIC Australia; 5 NorthWestern Mental Health, Parkville, VIC Australia; 6 Southern Synergy, Faculty of Medicine, Nursing and Health Science, Monash University, Clayton, VIC Australia; 7 Dept of Psychiatry, Monash University, Peninsula Mental Health Service, Frankston, VIC Australia; 8 School of Allied Health, Human Services and Sport, La Trobe University , Bundoora, VIC Australia

**Keywords:** Mental health service, Sub-acute, Residential unit, Routinely collected data, Consumer profile

## Abstract

**Background:**

There is an emerging international literature demonstrating clinical and cost-effectiveness of sub-acute residential mental health services. To date, however, there is limited information on the profile of consumers accessing these models of care. This study aimed to understand the profile of the population served by adult sub-acute residential mental health services in Victoria, Australia (known as Prevention and Recovery Care; PARC) and to compare PARC service consumers with consumers admitted to psychiatric inpatient units within public hospitals.

**Method:**

Using 5 years (2012–2016) of a state-wide database of routinely collected individual level mental health service data, we describe the socio-demographic and clinical profile of PARC service consumers compared to consumers of psychiatric inpatient units including for primary diagnosis and illness severity. Using admissions as the unit of analysis, we identify the characteristics that distinguish PARC service admissions from psychiatric inpatient admissions. We also examine and compare length of stay for the different admission types.

**Results:**

We analysed 78,264 admissions representing 34,906 individuals. The profile of PARC service consumers differed from those admitted to inpatient units including for sex, age, diagnosis and illness severity. The odds of an admission being to a PARC service was associated with several socio-demographic and clinical characteristics. Being male or in the youngest age grouping (< 20 years) significantly reduced the odds of admission to PARC services. The presence of primary diagnoses of schizophrenia and related disorders, mood, anxiety or personality disorders, all significantly increased the odds of admission to PARC services. Predictors of length of stay were consistent across PARC and inpatient admission types.

**Conclusions:**

Our findings suggest PARC services may serve an overlapping but distinguishably different consumer group than inpatient psychiatric units. Future research on sub-acute mental health services should be cognizant of these consumer differences, particularly when assessing the long-term effectiveness of this service option.

## Background

In 2003, the state of Victoria in Australia rolled out a new publicly funded sub-acute residential mental health service model known as Prevention and Recovery Care (PARC) services. PARC services, located in the community, offer sub-acute residential care to consumers with serious mental illness who require additional support than would usually be available from community mental health services. They have a strong emphasis on integrating clinical and personal recovery-oriented care, with a commitment to greater consumer involvement and least restrictive practices [1–6]. Similar to sub-acute residential services in other states in Australia and in some other high-income countries, PARC services in Victoria play an increasingly important role in the mental health care continuum, supporting consumers to transition from psychiatric inpatient units back into the community (referred to as ‘step-down’ care) and to prevent or substitute hospitalisation (‘step-up’ care).

PARC services operate as relatively small units with up to ten beds, offering short-term care (usually between 7 and 28 days) in a home-like environment. The majority are mixed gender and serve an adult population although more recently women only and youth PARC services have been established. They are run as a partnership between non-government agencies (known in Victoria as Mental Health Community Support Services; MHCSS) and local clinical mental health services. The clinical services provide assessment and treatment interventions, while the MHCCS provide 24-h residential services as well as assessment, treatment, psychosocial support to individuals and group activities. The service delivery model, informed by a personal recovery framework, is guided by the state government’s adult PARC services framework and operational guidelines [1, 7], described more fully elsewhere [7].

Studies exploring consumer experiences of the Victorian PARC service model [2] or their equivalents in other states [3–6] (mostly uncontrolled studies or studies limited by small sample sizes) have provided some information about how these services are operating at the local level. For example, a quasi-experimental study of crisis housing for patients with severe and persistent mental illness in Queensland demonstrated cost savings due to reduced acute psychiatric bed-days. However, these patients also experienced greater illness acuity and higher emergency department and inpatient admissions after the index episode compared with controls; it was unclear whether this could be explained by confounding or was a program effect [3]. A survey and interviews with clients of a step-up/step-down service in the Australian Capital Territory (ACT) reported improvements in consumer symptoms and functioning and mostly positive ratings of the service regarding the provision of a recovery-based program [4, 5]. Similarly, a mixed methods study combining an audit of medical files and interviews with consumers found that PARC services promoted recovery, improved outcomes and reduced subsequent admissions to inpatient psychiatric units [2]. In contrast to the findings in the ACT study, a recent Victorian Government report found no reduction in psychiatric hospital admission rates in areas with an established PARC service [8]. However, Victoria has the lowest number of acute beds per capita in Australia which may influence the extent to which admission rates are affected by other service options.

Although some of these early findings are promising, both international and Australian reviews of sub-acute residential services have concluded that studies are too few and of insufficient quality to evaluate effectiveness [9, 10]. One systematic review of residential alternatives to acute hospital admission included community-based services such as crisis housing, community mental health centre beds and adult family placements and identified only six moderate quality studies [9]. The review concluded that community-based alternatives were equivalent or better than standard inpatient care for symptomatic outcomes and consumer satisfaction. Cost-effectiveness also favoured community-based alternatives. While these findings are echoed elsewhere [10, 11], much of the existing research is limited by varied and poor definitions of what constitutes community-based alternatives; a paucity of long-term follow-up studies; poor descriptions of treatments and lack of detail about the consumer groups most likely to benefit [9, 10, 12, 13].

So there remain important but unanswered questions about what sub-acute residential care adds to existing mental health services [13]. A recent review specifically noted that studies exploring effectiveness and efficiency of residential sub-acute services rarely provide sufficiently detailed demographic data to understand the population served by these models of care [10]. We sought to overcome some of the limitations of earlier studies, particularly small sample sizes and inadequate descriptions of the residential sub-acute service and population served, by gathering detailed demographic and clinical data to understand the profile of the adult population served by PARC services and to compare PARC service consumers with those admitted to psychiatric units within public hospitals.

Drawing on a state-wide database of routinely collected individual level mental health service data, our specific aims were to: (1) describe the characteristics of PARC service consumers and compare these to consumers of inpatient services; (2) describe the characteristics of admissions, exploring differences between adult PARC service admissions and psychiatric adult inpatient unit admissions; and (3) examine length of stay among PARC service consumers, and for context, examine this also for admitted inpatients.

## Methods

### Study setting

In Victoria, state-funded public mental health services for adults are based on catchment areas, each comprising an acute hospital inpatient unit, a PARC service and community mental health services. Psychiatric inpatient units are located at the area general hospital, typically comprising 20–30 beds. Their primary role is provision of brief involuntary and voluntary admissions for symptom stabilisation and crisis resolution prior to referral for continuing care to community mental health, general practice or private services. Through the study period the Victoria population grew by 12% from 5.6 million to 6.2 million [14]. In that time, combined public sector bed provision in Victoria increased by 11% from 1241 (FYR 2011–2012) to 1376 (FYR 2016–2017) [15].

At the time of this study, the number of PARC services in Victoria had grown to 23, including 20 adult and three youth services (for consumers aged 16–25 years). The adult PARC services include a women’s only service and an extended stay service, where the expected stay is up to 6 months. Some short stay sub-acute services may also allocate one or more beds as an extended stay option. All PARC services have a system to assess suitability and prioritise referrals for admission. This process is not standardised across PARC services and there is variation in the process of entry [7]. Acceptance to a PARC service is determined jointly by the clinical and MHCCS provider following referral from the inpatient or community mental health team. This study focuses on the 19 adult sub-acute PARC services operating when this study commenced that routinely offer a maximum length of stay of 28 days (thus excluding the extended stay adult PARC), and the 19 inpatient units in the same catchments.

### Data source and sample

Following ethics approval from the University of Melbourne Human Research Ethics Committee (1648205), a deidentified dataset of relevant variables from the Client Management Interface/Operational Datastore (CMI/ODS) administered by the Victorian Government Department of Health and Human Services (DHHS) was provided to the researchers. The CMI/ODS is an electronic data system that records socio-demographic and diagnostic information of all consumers admitted to state-funded public mental health services in Victoria, Australia, as well as detailed information on service use. The CMI/ODS is primarily used for administration purposes in the sense that it records each contact with a consumer by a service provider.

The dataset included select variables for all individuals with an adult PARC service or acute hospital inpatient admission for the 5 years from 1 January 2012 to 31 December 2016. Although all 19 adult sub-acute PARC services were included in the dataset, the years of data available reflect the period in which the different services were in operation. For five PARC services there was less than 5 years of admissions data due to time since establishment. For one additional service only 1 year of data were available (2012) due to an administrative error in which the program type was miscoded in the subsequent 4 years. Additionally, records of admissions were excluded if a current admission had not ended at the end of the study period (31 December 2016) or for duplicate records.

Although we focus on adult mental health services, there was a small percentage of admissions to adult services for individuals falling outside of the 16 to 64 age range. We retained these cases, as their inclusion better reflect the population that typically use adult mental health services in the state. We did, however, exclude a small number of consumers who had been admitted to inpatient units as adults with previous admissions to youth PARC services. As one of our key variables of interest was illness severity as measured by the Health of the Nation Outcomes Scale (HoNOS) [16], we excluded admissions without a completed HoNOS. Our final analytic sample, therefore, consisted of 78,264 admissions representing 34,906 individuals.

### Measures

Individuals were categorised into three mutually exclusive groups based on all their admissions within the study period: 1) admission to adult PARC services only, 2) admission to acute adult inpatient psychiatric units only, or 3) any combination of the two. Admissions to PARC services were classified as ‘step down’ from an inpatient unit if the admission was listed in the routinely collected data as a ‘Transfer from Public Mental Health Inpatient Service,’ or if they had been discharged from an inpatient unit within the week prior to their PARC service admission. This timeframe is typical for PARC services to assess and admit consumers referred at the end of an inpatient stay. Admissions to PARC services were classified as ‘step up’ if referral was listed as from a community mental health service.

Socio-demographic variables included sex, age (coded as: < 20 years, 21–30 years, 31–40 years, 41–50 years, 51–60 years, > 61 years) and primary language spoken at home. Clinical information included primary diagnosis (according to ICD-10 codes), illness severity as assessed by the 12-item HoNOS, length of stay (LOS) in days and legal status at the time of admission. As individuals can have multiple primary diagnoses recorded per admission (interquartile range [IQR] 1–2, maximum 14), in addition to their non-primary diagnoses (maximum seven on any 1 day), we elected to include all primary diagnoses in the analyses. Diagnoses of organic disorders, including symptomatic mental disorders (ICD-10 codes F00-F09) and unspecified mental disorders (ICD-10 code F99) were dropped due to small numbers. Diagnoses of mental retardation, disorders of psychological development, behavioural and emotional disorders with onset usually occurring in childhood and adolescence (ICD-10 codes F70 – F98) were collapsed into a category hereafter referred to as ‘developmental disorders.’ Data on legal status at the time of admission accounted for changes that occurred midway through the study period to state-based legislation that governs involuntary admissions and community treatment orders.

Illness severity was assessed using the HoNOS [16]. The HoNOS is a measure of mental health and social functioning of individuals that is mandated as an outcome measure in most public mental health service settings in Australia. The scale contains 12 items divided into four subscales measuring behaviour, impairment, symptoms and social functioning. With item scores ranging from 0 (no problem) to 4 (severe to very severe problem), the HoNOS can be interpreted at the item, subscale or total score level. It is administered routinely at admission, review, transfer and discharge and may be completed multiple times during an inpatient stay and when the person is living in the community. For the purposes of this study, we used HoNOS scores at each admission or the last available score up to 2 months prior to admission. For each analysis using the HoNOS, subscale scores were checked for consistency using Cronbach’s alpha. If results showed inconsistency between items in a subscale (using the customary minimum cut-off of 0.7 [17]), individual items were used in their place.

### Statistical analysis

We describe the socio-demographic and clinical characteristics of consumers at their first admission according to admission type (PARC service only, inpatient only, or both PARC and inpatient). Characteristics examined were sex, age group, legal status, primary language and the seven primary diagnoses described above. Differences were identified using a Chi square test, and where a significant difference was observed, adjusted standardised residuals to probe where the observed and expected cell counts differed. Residuals with a value less than − 3 were used to identify cells with a lower than expected count; residuals greater than 3 flagged those with a higher than expected count [18].

Using the admission episode as the unit of analysis, we then identified the characteristics that distinguish PARC service admissions from inpatient admissions. We did this descriptively (calculating counts and proportions) and then used logistic regression to estimate the odds of any given admission being to a PARC service adjusting for covariates. For this analysis, the outcome was a binary coded variable with a value “1” if the admission was a PARC service admission and a value of “0” if it was an inpatient admission. The predictor variables were sex, age group, primary diagnosis, and binary coded variables representing a high HoNOS items/subscale scores (0 to 3 vs. ≥3 for items and subscales). We present the results on the exponential scale as odds ratios. Because individuals could have multiple admissions, we used cluster-adjusted robust standard errors to calculate 95% confidence intervals (CI). Finally, to determine predictors of length of stay (LOS), we used negative binomial regression using the same set of predictor variables as the logistic regression model, except for the HoNOS items, which were entered as a continuous variable on their original metric (score of 0 to 4) and admission number (also entered as a continuous variable). Cluster adjusted standard errors were again used. Results are reported as rate ratios. Models were run separately for PARC service admissions and inpatient admissions.

## Results

### Characteristics of consumers

Of the 34,906 individuals in the study sample, 4% (*n* = 1588) were only admitted to a PARC service and 80% (*n* = 27,755) were only admitted to an adult inpatient unit within the five-year study period. The remaining were admitted to a combination of the two service types (16%; *n* = 5566).

There were differences between these three groups on a number of demographic and clinical characteristics (Table 1). Of those only admitted to PARC services, 61.8% were women (compared to 43.8% for those only ever admitted to an acute inpatient unit and 53.4% for those admitted to both services). Consumers only ever admitted to a PARC service were more likely to be aged ≥50 years than those admitted to inpatient units only (25% vs. 17%); whereas those admitted to inpatient units only were more likely to be age ≤ 30 years (34% vs 23%). Those only admitted to inpatient units were more likely than those admitted to PARC services to have been diagnosed with schizophrenia and related disorders or substance use disorders (25.1% vs. 20.1%). PARC service only consumers were more likely to have a diagnosis of mood, anxiety and personality disorders than inpatient only consumers (45.4% vs. 19.0, 23.0% vs. 10.4, 10.9% vs. 5.5%, respectively). Eight percent of those only admitted to an inpatient unit were for substance use disorders (cf. 4.6% of PARC only consumers).

**Table 1:**
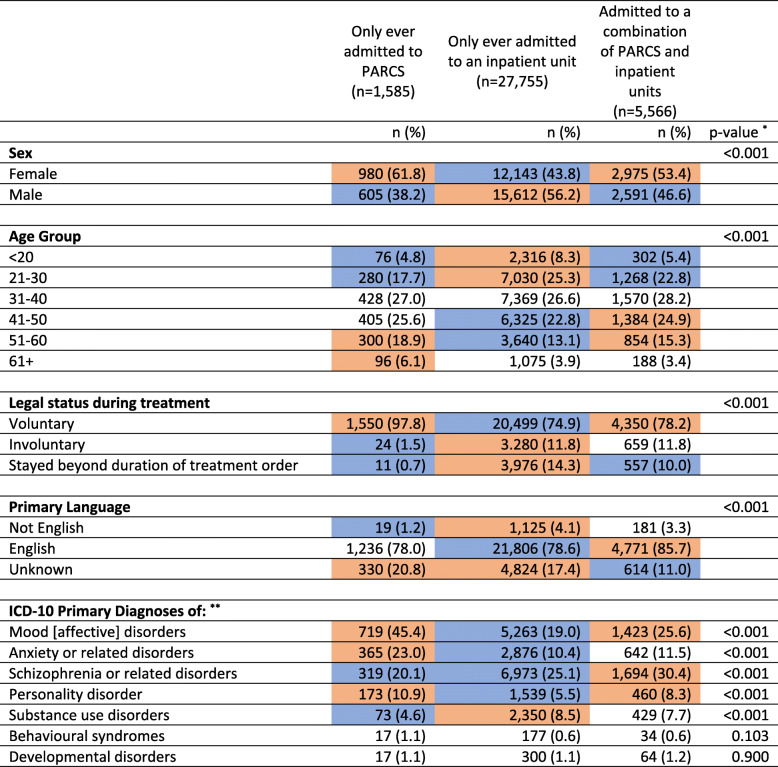
Demographics of consumers at their first admission, by admission type history

*P-value calculated using Chi-squared test. Orange represent adjusted standardised residuals ≥3 which indicate a greater than expected number of cases in that cell. Blue represent adjusted standardised residuals with ≤ −3 which indicate a lower than expected number of cases in that cell. All others represent residuals between these values and indicate observed and expected do not differ

**Diagnoses are not mutually exclusive. As such, *p*-values are calculated for each diagnosis

At their first admission in the study period, the majority of consumers only ever admitted to a PARC service had entered and exited treatment voluntarily (97.8%). For those only ever admitted to acute inpatient units, 11.8% were under a compulsory treatment order for the duration of their index admission. Fourteen per cent entered on a compulsory treatment order but changed to voluntary status prior to discharge (with a median of 3 days spent in the inpatient unit after the order expired; IQR: 1–7 days).

**Fig. 1:**
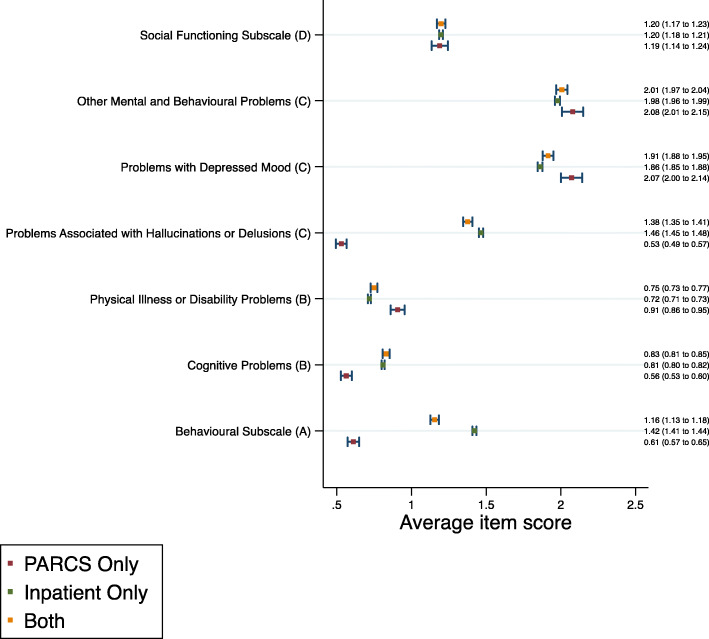
HoNOS subscale/item scores at first admission, by admission type. Admission type

Results also indicated differences in baseline HoNOS scores. For the behavioural subscale, those who were only ever admitted to PARC services had lower scores than the other two groups (Fig. 1). The social functioning subscale showed no difference for consumers of PARC services and inpatient units. Cronbach’s alpha for the impairment and symptoms HoNOS subscales fell below the acceptable criterion for reliability (alpha = 0.7), suggesting that the items were measuring different constructs, and were analysed as individual items. Those who were only ever admitted to PARC services scored higher on three HoNOS items relating to physical illness and disability, depressed mood and other mental and behavioural problems than those admitted to inpatient units only, but had lower average scores for the cognitive problems item. High scores on the item measuring problems associated with hallucinations and delusions were more common for those who were only ever admitted to inpatient units or who were admitted to both services.

### Characteristics of admissions

Of the total 78,264 admissions in the study sample, 14% (*n* = 10,835) were to a PARC service. For all PARC service admissions in the study period, 38% (*n* = 4073) were categorised as ‘step-down’, indicating transfers from hospital-based psychiatric inpatient units to PARC services. There was however variation in the proportion of admissions categorised as ‘step-down’ by individual PARC services, ranging from 25 to 60%.

The logistic regression model showed that the odds of an admission being to a PARC service was associated with several socio-demographic and clinical characteristics of consumers (Table 2). Males had lower odds of being a PARC service admission than females (OR = 0.71; 95% CI = 0.67–0.76). In general, the odds of being admitted to a PARC service increased with age (e.g., OR for 21–30 years vs. ≤20 years was 1.36, 95% CI 1.17 to 1.58). The presence of a primary diagnoses of schizophrenia and related disorders (OR = 2.32, 95% CI 2.17 to 2.48), a mood disorder (OR = 2.79, 2.63 to 2.96), an anxiety disorder (OR = 2.09, 95% CI 1.95 to 2.24), a personality disorder (OR = 1.53, 95% CI 1.38 to 1.68) or a developmental disorder (OR = 1.29, 95% CI 1.04 to 1.61) were all associated with increased odds of admission to a PARC service. No differences were observed for people with a diagnoses of substance use disorder (OR = 1.04, 95% CI 0.97 to 1.13) or behavioural syndromes (OR = 1.09, 95% CI 0.85 to 1.42).

**Table 2:** Demographics of users for all admissions, and odds of admission to PARCS, *n* = 78,264

	PARCS (*n* = 10,835)	Inpatient (*n* = 67,429)	Adjusted Odds Ratio for being admitted to PARC service rather than an adult acute inpatient ward and	*p*-value
	n (%)	n (%)	OR (95% CI)	
**Sex**				
Female (ref.)	6213 (57.3)	31,216 (46.3)	1.00	
Male	46,22 (42.7)	36,213 (53.7)	0.71 (0.67, 0.76)	< 0.001
**Age Group**				< 0.001
< 20 (ref.)	454 (4.2)	4582 (6.8)	1.00	
21–30	2307 (21.3)	17,380 (25.8)	1.36 (1.17, 1.58)	
31–40	3023 (27.9)	19,193 (28.5)	1.62 (1.39, 1.88)	
41–50	2788 (25.7)	15,257 (22.6)	1.79 (1.54, 2.08)	
51–60	1821 (16.8)	8718 (12.9)	1.84 (1.57, 2.16)	
61+	442 (4.1)	2299 (3.4)	1.65 (1.36, 1.99)	
**ICD 10 Primary Diagnoses of:**^**a**^				
Schizophrenia or related disorders	4552 (42.0)	27,113 (40.2)	2.32 (2.17, 2.48)	< 0.001
Mood [affective] disorders	4371 (40.3)	15,295 (22.7)	2.79 (2.63, 2.96)	< 0.001
Anxiety or related disorders	2135 (19.7)	7548 (11.2)	2.09 (1.95, 2.24)	< 0.001
Personality disorder	1950 (18.0)	9357 (13.9)	1.53 (1.38, 1.68)	< 0.001
Substance use disorders	1477 (13.6)	9790 (14.5)	1.04 (0.97, 1.13)	0.257
Behavioural syndromes	102 (0.9)	526 (0.8)	1.09 (0.85, 1.42)	0.493
Developmental disorders	219 (2.0)	1136 (1.7)	1.29 (1.04, 1.61)	0.021
**High HoNOS Items/Subscale Scores**^**b**^				
Other Mental and Behavioural Problems (i)	3124 (28.8)	27,274 (40.5)	0.81 (0.81, 0.90)	< 0.001
Problems with depressed mood (i)	2500 (23.1)	22,877 (33.9)	0.58 (0.55, 0.62)	< 0.001
Problems associated with hallucinations or delusions (i)	1348 (12.4)	23,173 (34.4)	0.31 (0.29, 0.34)	< 0.001
Social Functioning Subscale (ss)	1229 (11.3)	11,736 (17.4)	1.03 (0.96, 1.11)	0.395
Physical Illness or Disability Problems (i)	987 (9.1)	7098 (10.5)	1.02 (0.92, 1.13)	0.688
Cognitive Problems (i)	452 (4.2)	8620 (12.8)	0.45 (0.41, 0.52)	< 0.001
Behavioural Subscale (ss)	310 (2.9)	14,467 (21.5)	0.15 (0.13, 0.17)	< 0.001

^a^Diagnoses are not mutually exclusive. Reference category is no such diagnosis

^b^High values are ≥3 on a 0–4 scale for both Items and Standardised Subscales. Reference category is a score < 3

(i) denotes HoNOS item

(ss) denotes HoNOS subscale

In terms of illness severity, no HoNOS items or subscale scores were associated with increased odds of being admitted to a PARC service, but the behavioural subscale (OR = 0.15, 95% CI 0.13 to 0.17), problems with hallucinations or delusions (OR = 0.31, 95% CI 0.29 to 0.34), cognitive problems (OR = 0.45, 95% CI 0.41 to 0.52), problems with depressed mood (OR = 0.58, 95% CI 0.55 to 0.62) and other mental and behavioural problems (OR = 0.81, 95% CI 0.81 to 0.90) were all associated with lower odds of being admitted to a PARC service.

### Factors affecting LOS

The average (median) LOS for an acute inpatient admission was 8 days (IQR of 3–17 days). PARC service admissions had a median of 16 days (IQR of 10–26 days). Using negative binomial regression to separately model LOS for PARC service and inpatient admissions, we found similarities and differences in socio-demographic and clinical predictors of LOS by admission type (Table 3). In comparison to women, LOS for men was shorter; 3% shorter for PARC service admissions (RR = 0.97, 95% CI 0.93 to 0.97) and 6% shorter for acute inpatient admissions (RR = 0.94, 95% CI 0.93 to 0.96). For PARC service admissions, LOS was 5% longer in the presence of ICD-10 primary diagnosis of substance use disorder (RR = 1.05, 95% CI 1.02 to 1.09) and 7% shorter in the presence of personality disorder (RR = 0.93, 95% CI 0.90 to 0.97). In terms of primary diagnoses, predictors of LOS were consistent across PARC and inpatient admission types for personality disorders (i.e., shorter stays), but inconsistent on all other diagnoses.

**Table 3:** Predictors of LOS in PARC Services and Inpatient Units

	PARCS (*n* = 10,835)		Inpatient (*n* = 67,429)	
	RR (95% CI)	*p*-value	RR (95% CI)	*p*-value
**Sex**				
Female (ref.)	1.00		1.00	
Male	0.97 (0.95, 1.00)	0.026	0.94 (0.93, 0.96)	< 0.001
**Age Group**		< 0.001		< 0.001
< 20 (ref.)	1.00		1.00	
21–30	1.22 (1.14, 1.30)		1.06 (1.02, 1.09)	
31–40	1.22 (1.15, 1.31)		1.12 (1.09, 1.16)	
41–50	1.26 (1.19, 1.35)		1.14 (1.10, 1.18)	
51–60	1.32 (1.23, 1.41)		1.30 (1.26, 1.35)	
61+	1.41 (1.29, 1.53)		1.37 (1.30, 1.43)	
**ICD-10 Primary Diagnoses of:**^**a**^				< 0.001
Substance use disorders	1.05 (1.02, 1.09)	< 0.001	0.97 (0.94, 0.99)	
Schizophrenia or related disorders	1.01 (0.99, 1.04)	0.690	1.36 (1.34, 1.39)	
Mood [affective] disorders	1.03 (1.00, 1.06)	0.046	1.18 (1.15, 1.20)	
Anxiety or related disorders	1.02 (0.99, 1.06)	0.140	0.84 (0.82, 0.86)	
Behavioural syndromes	1.14 (1.00, 1.29)	0.042	1.39 (1.28, 1.50)	
Personality disorder	0.93 (0.90, 0.97)	< 0.001	0.79 (0.77, 0.81)	
Developmental disorders	0.98 (0.00, 1.07)	0.607	1.13 (1.07, 1.19)	
**Admission number**	0.99 (0.99, 0.99)	< 0.001	0.99 (0.98–0.99)	< 0.001
**HoNOS Items**^**b**^				
Overactive, aggressive or disruptive behaviour	0.94 (0.92, 0.95)	< 0.001	1.05 (1.04, 1.06)	< 0.001
Non-accidental self-injury	1.00 (0.99, 1.02)	0.714	0.95 (0.95, 0.96)	< 0.001
Problem-drinking or drug taking	0.96 (0.95, 0.97)	< 0.001	0.94 (0.94, 0.95)	< 0.001
Cognitive problems	0.99 (0.98, 1.00)	0.190	1.06 (1.06, 1.07)	< 0.001
Physical illness or disability problems	0.98 (0.97, 0.99)	0.001	1.01 (1.01, 1.02)	< 0.001
Problems associated with hallucinations or delusions	1.05 (1.04, 1.06)	< 0.001	1.10 (1.09, 1.10)	< 0.001
Problems with depressed mood	0.99 (0.98, 1.00)	0.056	0.96 (0.95, 0.97)	< 0.001
Other mental and behavioural problems	1.01 (1.00, 1.02)	0.022	0.99 (0.98, 0.99)	< 0.001
Problems with relationships	0.99 (0.98, 1.00)	0.097	0.97 (0.96, 0.98)	< 0.001
Problems with activities of daily living	1.04 (1.02, 1.05)	< 0.001	1.05 (1.04, 1.06)	< 0.001
Problems with living conditions	1.04 (1.03, 1.05)	< 0.001	1.04 (1.03, 1.04)	< 0.001
Problems with occupation and activities	0.98 (0.97, 0.99)	0.004	0.99 (0.99, 1.00)	0.022

^a^Diagnoses are not mutually exclusive. Reference category is no such diagnosis. ^b^Reference category is “no” to the HONOS item

Some items on the HoNOS were also associated with LOS. For PARC service admissions, LOS was longer for consumers with diagnoses of hallucinations and delusions (RR = 1.05, 95% CI 1.04 to 1.06), problems with daily living (RR = 1.04, 95% CI 1.02 to 1.05) and problems with living conditions (RR = 1.04, 95% CI 1.03 to 1.05), while problematic drinking or drug taking (RR = 0.96, 95% CI 0.95 to 0.97) and aggressive, overactive or disruptive behaviour problems (RR = 0.94, 95% CI 0.92 to 0.95) were associated with shorter stays. This trend was broadly consistent across both admission types except for aggressive, overactive or disruptive behaviour which was associated with shorter stays in inpatient units (RR = 1.05, 95% CI 1.04 to 1.06), but with longer stays in PARC services.

## Discussion

The current study used a routinely collected state-wide data collection to assess the characteristics of consumers admitted to adult mental health services, exploring differences between admissions to PARC services in comparison with hospital-based psychiatric inpatient units. Only a small proportion of consumers accessing care in the five-year study period were admitted to PARC services only, with most consumers also accessing inpatient units. Despite some overlap, the socio-demographic and clinical profile of PARC service consumers differed from those only ever admitted to acute inpatient units. Consistent with prior research, PARC service consumers were more likely to be female, voluntary patients with primary diagnoses of mood, anxiety and/or personality disorders [13, 19]. PARC service consumers were more likely to present with significantly lower scores on the HoNOS behavioural subscale indicating they were less likely to be presenting with problems related to aggression, self-harm and substance use. These findings were confirmed by the admissions data, and are consistent with a recent commentary suggesting sub-acute models of care may serve a population with a lower behavioural risk profile than served by inpatient units [20].

Although results indicated that less complex and/or less severe illness was more likely among PARC service admissions, other indicators suggest that those admitted to PARC services do have specific and complex needs. This includes, for example, a greater likelihood of high scores on the HoNOS physical illness or disability problems item and on the social functioning subscale. While there are a range of tensions expressed in the literature regarding the purpose and intent of new models of sub-acute community-based residential care, including whether they can reduce demand on specialised psychiatric units [20, 21], the different consumer profiles suggest these services are providing a complementary rather than a substitute mental health service option for a specific group of consumers. Greater clarity about the needs of subgroups of PARC service consumers, such as those transitioning from hospital-based care (step-down), or those with non-psychotic disorder diagnoses, may assist both in service planning and in guiding expectations for outcomes for sub-acute services.

Similar to our results, other researchers have noted a high level of social problems among consumers entering sub-acute residential mental health services [5]. Further, those with a longer LOS were more likely to have substance use issues and be identified as having aggressive, overactive or disruptive behaviour. The longer LOS at PARC services may be providing an opportunity to provide more holistic care (consistent with the personal recovery model) rather than a narrow focus on short-term symptom reduction or substance intoxication or withdrawal as appears to be occurring in inpatient units. Prediction of shorter LOS in either setting by ICD-10 primary diagnosis of personality disorder has been reported elsewhere [22]. Our results demonstrate that personality disorder is common among PARC service consumers and is characterised by relatively short LOS. This suggests that PARC services are providing care for consumers with personality disorder that closely parallels inpatient units, but with the potential to act as alternative location for a short residential admission.

Results showed the typical care pathway to PARC services was from community-based care, with most admissions categorised as ‘step-up’. Although there was variation by individual service in terms of the proportion of ‘step-up’ and ‘step-down’ referrals, the finding that most admissions were initiated in the community indicates that PARC services are providing an avenue for mental health service consumers to access a higher level of intensity of care. We cannot say from this data set however for what proportion this may be in response to an escalation in symptoms, early signs of relapse, break down in social supports or other reasons. It is possible that not all ‘step-up’ admissions were intended to prevent immediate hospitalisation: the function may have been early intervention. For example, some PARC service admissions may have enabled access to increased intensity of psychosocial and biomedical interventions for consumers with psychotic disorders for early intervention in a relapse prodrome period; others may have been brief admissions for people living with a diagnosis of personality disorder as part of a program of community based treatment. Whether sub-acute models are seen simply as a substitute for a proportion of inpatient admissions or as providing further community-based service options has been previously raised in the literature [12, 20]. The latter interpretation is consistent with a qualitative study with consumers of a step-up/step-down service located in the ACT, which suggested that ‘step-up’ clients saw the service as an intensive intervention and a place of treatment, therapy and learning [4].

Study findings should be interpreted in light of a number of limitations. We used 5 years of mental health service data categorising consumers into admission type based on all admissions within this period. We cannot, however, comment on consumer admissions prior to 2012. We also note that the study covers a period in which PARC services were expanding in the state and, as such, data were not available for all adult PARC services for all 5 years. The DHHS additionally excluded data for all but 1 year for one PARC service because of an administrative coding error. We also excluded from analyses the extended adult PARC service (with expected stays of up to 6 months) because of the potential to artificially distort findings in terms of clinical indicators, such as LOS. It is also acknowledged that these data only cover state-funded services and thus exclude care provided to consumers through private specialists. Additionally, admissions data could only be dichotomised into either ‘step-up’ or ‘step-down’ without further information available to differentiate on intention (i.e., admissions as a more intensive alternative to community-based care).

Despite these limitations, the study fills an important gap in the literature; its key strength being the use of routinely collected individual level mental health service data. Although collected for administrative and clinical purposes and not specifically for research, it provides the largest data capture on mental health service use in the state and allowed us to describe in detail the socio-demographic and clinical profile of PARC service consumers in comparison to those admitted to inpatient psychiatric care.

## Conclusions

In Australia, as in many other parts of the world, acute inpatient care remains an important element of service response for people experiencing severe mental illness. Yet, in policy and practice there is an increasing emphasis on providing alternatives to hospital-based care, particularly service models with an explicit focus on personal recovery. PARC services, first introduced in Victoria over 15 years ago, have expanded in the state and this study indicates they are serving a consumer group that shares many of the same characteristics as consumers admitted to inpatient units, but with some important differences. These similarities and differences have implications for the role PARC services are playing in the mental health service network. At one level, they appear to be filling an important service gap between hospital inpatient and community-based care by providing ‘step-up’ and ‘step-down’ care pathways. Study findings also suggest they may be offering a new option, that could be described as more intensive care for consumers with additional needs that are unlikely to be met by community care alone.

Our study adds to the growing body of literature on residential sub-acute care by profiling a large population typically served by these models of care. Evidence presented here suggests PARC services may serve an overlapping but distinguishably different consumer group than inpatient psychiatric units. Future research should be cognizant of these consumer differences, particularly when assessing the roles and long-term effectiveness of this service option.

## Data Availability

The data that support the findings of this study are available from the Victorian Government Department of Health and Human Services, but restrictions apply to their availability which were used under license for the current study, and so are not publicly available. However, data may be available from the authors upon reasonable request, and with permission of the Victorian Government.

## Abbreviations

ACT: Australian Capital Territory
CMI/ODS: Client Management Interface/Operational Datastore
DHHS: Department of Health and Human Services
HoNOS: Health of the Nation Outcomes Score
ICD-10: International Classification of Diseases, 10th revision
LOS: Length of stay
MHCSS: Mental Health Community Support Services
PARC: Prevention and Recovery Care
